# Spacetime in the brain: rapid brain network reorganization in visual processing and recovery

**DOI:** 10.1038/s41598-021-96971-8

**Published:** 2021-09-09

**Authors:** Zheng Wu, Bernhard A. Sabel

**Affiliations:** 1 Institute of Medical Psychology, Medical Faculty, Otto-von-Guericke University of Magdeburg, Magdeburg, Germany; 2 Data and Knowledge Engineering Group, Faculty of Computer Science, Otto-von-Guericke University of Magdeburg, Magdeburg, Germany

**Keywords:** Optic nerve diseases, Visual system

## Abstract

Functional connectivity networks (FCN) are the physiological basis of brain synchronization to integrating neural activity. They are not rigid but can reorganize under pathological conditions or during mental or behavioral states. However, because mental acts can be very fast, like the blink of an eye, we now used the visual system as a model to explore rapid FCN reorganization and its functional impact in normal, abnormal and post treatment vision. EEG-recordings were time-locked to visual stimulus presentation; graph analysis of neurophysiological oscillations were used to characterize millisecond FCN dynamics in healthy subjects and in patients with optic nerve damage before and after neuromodulation with alternating currents stimulation and were correlated with visual performance. We showed that rapid and transient FCN synchronization patterns in humans can evolve and dissolve in millisecond speed during visual processing. This rapid FCN reorganization is functionally relevant because disruption and recovery after treatment in optic nerve patients correlated with impaired and recovered visual performance, respectively. Because FCN hub and node interactions can evolve and dissolve in millisecond speed to manage spatial and temporal neural synchronization during visual processing and recovery, we propose “Brain Spacetime” as a fundamental principle of the human mind not only in visual cognition but also in vision restoration.

## Introduction

For centuries neuroscientists have explored how circumscribed brain centers support mental functions such as vision, language or cognition^1^. This “localizationist” approach focused on how *local* neuronal cell assemblies or brain regions control specific sub-functions. But this localization cannot fully explain different behavioral phenomena such as rapid plasticity in normal learning^2^, transmodal plasticity^3^, recovery of function^4^, or receptive field plasticity following visual system damage which can happen even within minutes^5^. It is the new network science which paves the way for a more *global* perspective of how neuronal information is integrated between regionally distributed *local* brain centers.

Functional connectivity network (FCN) analyses have become popular in recent years to unravel the spatial and temporal organization of ***local*** and ***global*** neural processing^6^. Typically, FCN analyses use resting-state data^7^, but rapid and transient changes of FCN (rFCN) were  not yet studied in the human brain on a millisecond scale. Yet, the maximum speed of FCN dynamics needs to be known, because FCN provide the physiological support for top-down stimulus processing and synchronization of sensory, motor and cognitive functions. The synchronous mode uses a gain approach, weighting the anatomical connections to generate effective interaction patterns, where the phase relation supports interactions between neuronal assemblies as a function of brain regions (space), time, and specific frequencies^8^. Although temporal dynamics of brain activities are often studied^9^, little is known how fast multiple brain regions are “bound” in time through phase synchronization in the brain’s FCN topological “workspace”^10^.

Compared to the 4–8 s BOLD response in magnetic resonance imaging (MRI), the EEG has an infinite time resolution, allowing us to explore if and how fast FCN can synchronize, including events that have no (measurable) energy consumption^6^. For example, EEG recordings show phase synchronized patterns and temporal brain dynamics during face perception tasks^11^, and it can display unique, dynamic patterns of FCN changes, for example in auditory and visual oddball tasks^12^, or during face and object matching tasks in autism spectrum conditions^13^.

While such studies confirm that individual brain regions can interact with each other in millisecond speed, rapid dynamics of whole brain FCN plasticity has not been explored. Yet, this is critical to fully understand how higher order “top down” cognitive influences can actively support, or interfere with, “bottom-up”, afferent input^14^, especially in fast mental acts or behavioral tasks which happen at a split of a second such as temporal discrimination (20–40 ms)^15^ or fast visual detection (150–200 ms).

Also cognitive processing requires rapid and transient dynamic reorganization of brain functional networks^16^, so that topological rearrangements can enable the synchronization and integration of neural processing during different cognitive modalities. But when neural synchronization is disturbed, this can lead to impairment or loss of functions. For example, optic nerve patients with vision loss show FCN disturbances in the resting state^17^, and with neuromodulation using repetitive transorbital alternating current stimulation (rtACS) they can be partially restored which induces vision recovery^18^. But it is unknown if, and to what extent, fast FCN reorganization exists and how this affects - or is affected by - damage.

To explored rapid FCN dynamics at millisecond resolution we now used our “event related network analysis” (ERNA)^16^ in patients with optic nerve damage. Here, similar to visual evoked potentials, EEG recordings are time-locked to visual stimulus onset, and subsequent FCN graph analysis can show how topological 3D-“*Space*” (individual brain regions) is linked with the 4th dimension of “*Time*”.

In the present study we hypothesized that patients with optic nerve damage suffer disturbances in rapid visual processing and that rtACS can modulate topological networks structures and induce vision restoration. To this end, we first determined brain regions with the highest level of synchrony (“hubs”) and functional connections between them before and after stimulus onset, comparing network dynamics in normal subjects and patients with vision loss at millisecond resolution. We next wished to learn if FCN metrics correlate with normal and abnormal visual performance. Finally, we investigated the effects of "neuromodulation" with rtACS to explore the functional relevance of fast FCN changes for "visual performance". Specifically, we hypothesized that (i) brain spacetime dynamics can be monitored by hubs and their connections at millisecond resolution, (ii) FCN can vary systematically in their strength, stability and dynamics, (iii) FCN dynamics are disorganized in patients with low vision, and (iv) they can be modulated with rtACS which correlates with vision recovery.

## Results

To characterize FCN, graph metrics were time-locked to visual stimulus onset and analyzed for each region of interest (ROI) on a time vector sub-divided in 8 ms time-windows (see “Methods”). Our graph metrics included global network topology measures, clustering coefficient (CC), characteristic path length (CPL), and small-worldness (SW). We also investigated the hub topography as characterized by hub scores (HS), and different node centrality metrics (weighted betweenness centrality, weighted closeness centrality, weighted degree).

### Brain Spacetime in normal subjects

**Figure 1:**
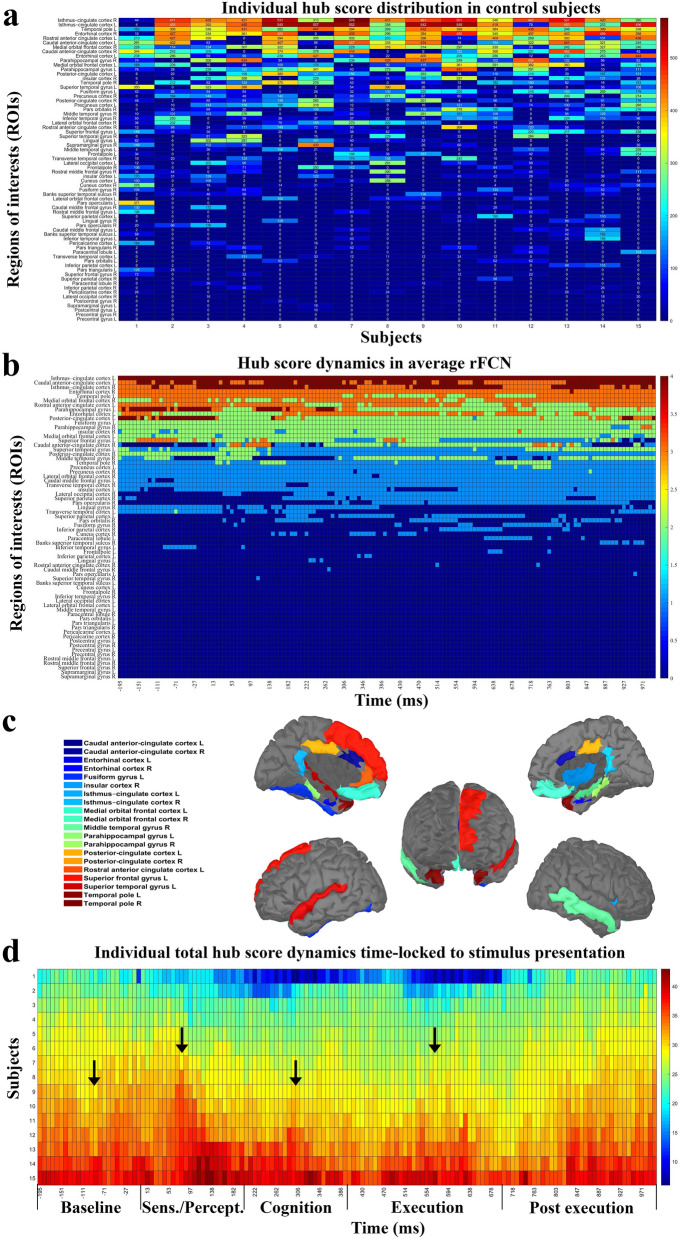
Hub score distribution and changes across time in high alpha FCN. (**a**) Hub score distribution in networks of individual control subjects during visual processing. Each value shows the sum hub score (0–4) during the recording epoch of − 200 to + 1000 ms for the respective brain areas. The regions are sorted from small to large. Control subjects varied in their individual hub localization, hub number and hub strength. (**b**) Transient hub score distribution of averaged healthy brain networks. Hub scores were computed for each region of interest (ROI) at each time point. If a region had a hub score ≥ 2 and a duration of ≥ 50 ms without interruption throughout the visual process, this node was identified as a hub. ROIs were sorted according to hub strength, differing in their hub (synchronization) strength and stability across time (scale shows averaged hub score 0–4 during visual processing). (**c**) In normal subjects we identified rFCN in 20 ROIs which were hubs in the high alpha-band during the visual task in cingulate (n = 7), temporal (n = 9) and frontal cortex (n = 3); and in the insular region (n = 1). (**d**) Total hub score changes across time for all control subjects for the 20 hubs. It shows phases of hub score strength fluctuations across time. To facilitate interpretation, FCN transients were divided into different functional stages of visual processing: “baseline”, from − 200 to 0 ms ([− 200, 0] ms), “sensation/perception” ([0, 200] ms), “cognition” ([200, 400] ms), “execution” ([400, 700) ms) and “post-execution” ([700, 1000] ms). Of note, peaks of hub strengths correspond to those typically found in evoked potential recordings (e.g. N100, P300).

In control subjects with normal vision 20 FCN ‘hubs’ could be identified in the high alpha band in the default mode network (DMN), attention network (AN), salience network (SN) or execution control network (ECN) (Fig. 1). Surprisingly, primary visual cortex and visual-association areas had no hubs. Hub distribution within individual FCN could change rapidly (Fig. 1a) and the averaged rapid FCN (Fig. 1b,c) were similar, but hub strength was quite different between individuals.

**Figure 2:**
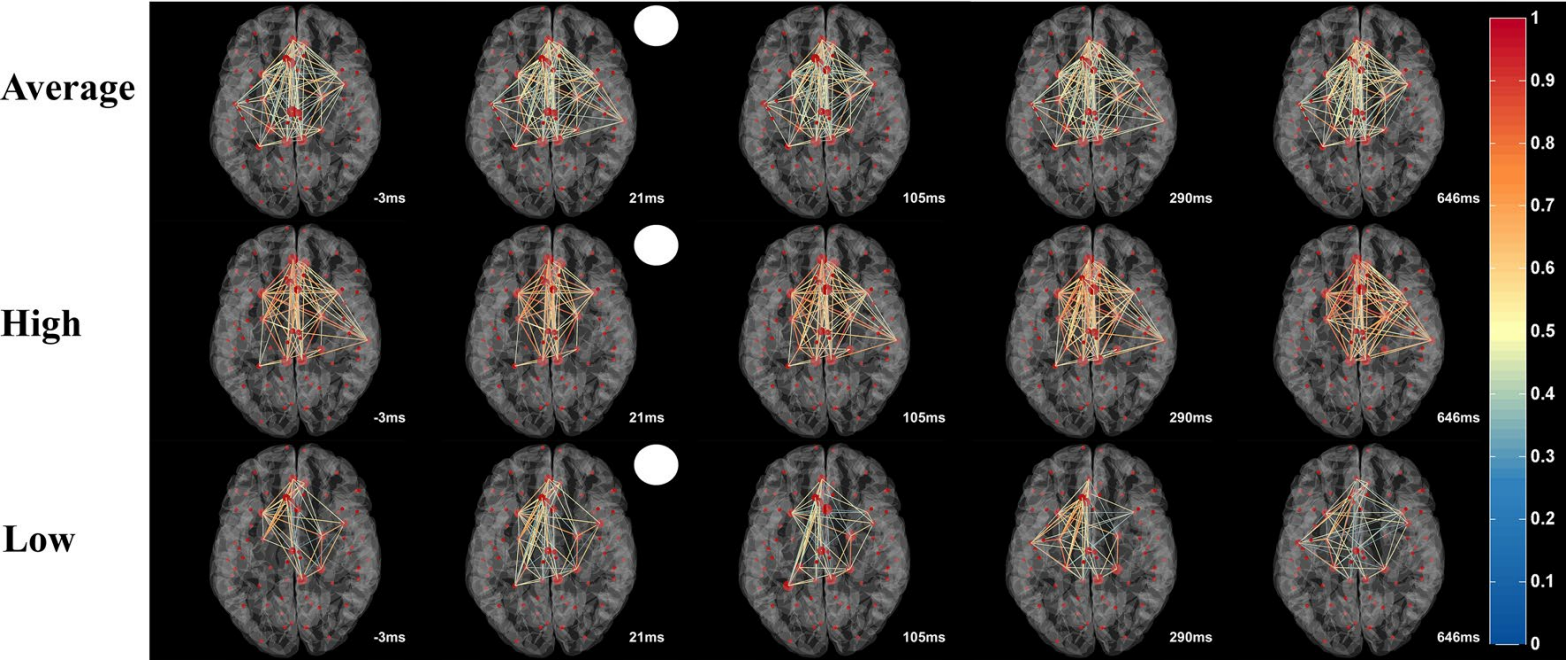
Imaging “Brain Spacetime” in visual processing. Transient network formations during visual processing displayed in time-slices as a function of visual stimulus onset (white circle) in averaged normal rFCN. Hubs (big red dots) represent ROIs with high synchronization (hub scores 2–4, displayed by hub size). Links between the hubs were color-plotted as a function of their weight from 0 to 1. The top row shows the averaged hub locations and their inter-hub connections in normal brains. The lower rows show two representative subjects with different levels of network synchronization (high and low). The real-time video displayed in the Supplementary Video S1 illustrate the rapid rFCN dynamics of hubs in 8 ms resolution.

Although overall hub-topography was relatively stable, hub-strength and connections between hubs were highly dynamic during the 1-s time vector following stimulus onset. Inter-hub connections changed their strength in tremendous (ms) speed (Fig. 2) which can be viewed in the Supplementary Video S1. As it shows, how hubs’ strength and connections rapidly evolve and/or dissolve. After stimulus onset, the average rFCN had more connections compared to baseline. Here, the phase synchronized strength increased at around 300 ms. At around 600 ms, when the response button was pressed, the connections were densely connected. This pattern suggests that rFCN dynamics are behaviorally meaningful. Yet, the fluctuations of total hub scores show a typical oscillation pattern with peaks that are comparable to those found in visual evoked potentials (VEPs) (such as N100 and P300) (Fig. 1d). To facilitate interpretation, FCN transients were divided into different functional stages of neural processing that were known from visual evoked potentials: “baseline”, from − 200 to 0 ms ([− 200, 0] ms), “sensation/perception” ([0, 200] ms), “cognition” ([200, 400] ms), “execution” ([400, 700] ms) and post-execution ([700, 1000] ms).

Rebustness of hub selection threshold was confirmed by comparing our results with 15% and 25% thresholds, both of which showed similar results.

### Brain Spacetime in patients

**Figure 3:**
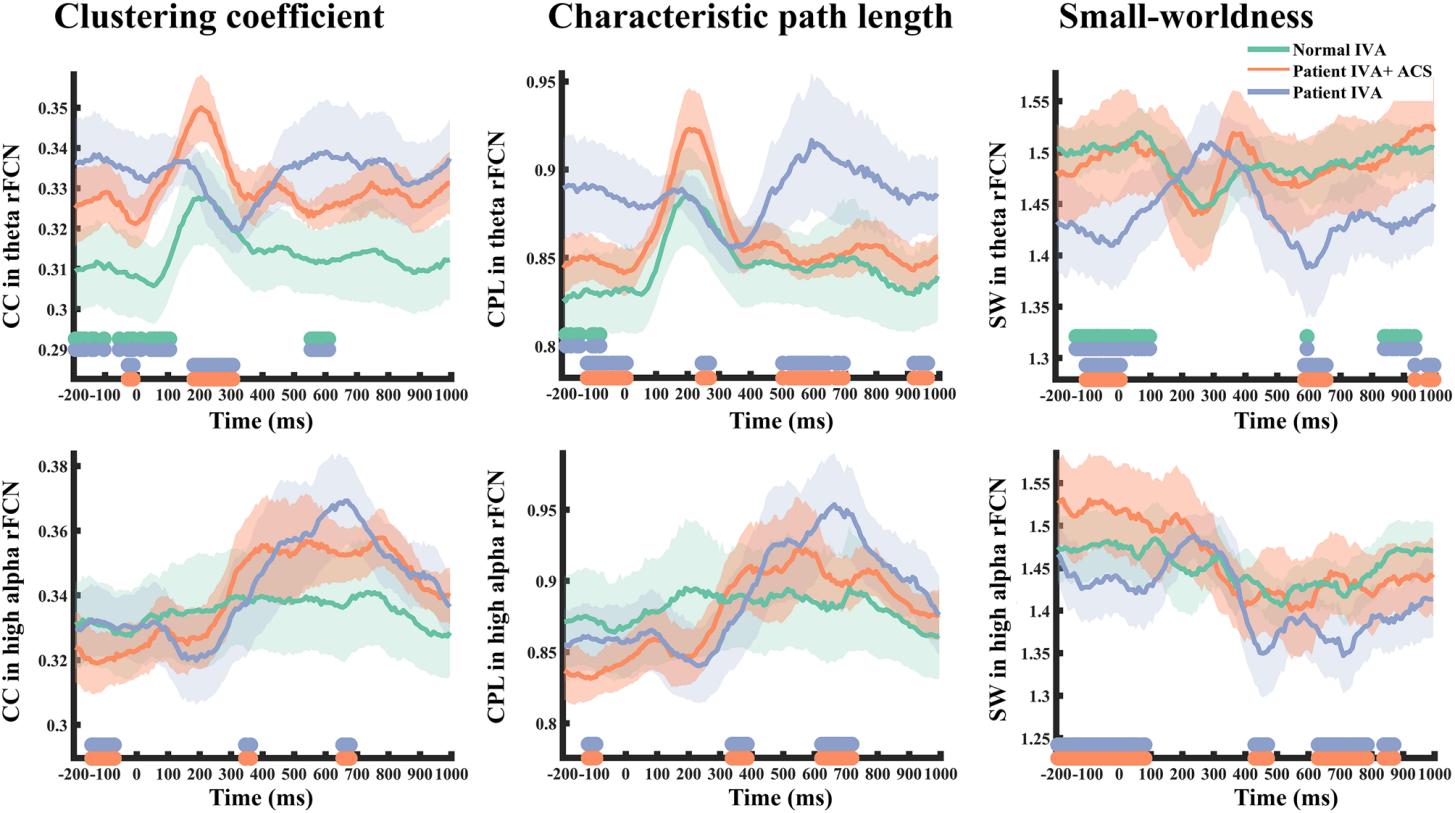
Transient dynamics of global rFCN parameters. Multiple global rFCN matrices change across time during visual processing in control subjects (green line) and patients with optic nerve damage pre- (blue) and post-rtACS treatment (red). The horizontal green–blue bars on the x-axis indicate time windows with significant differences between controls and patients; orange–blue bars mark significant differences of pre- vs. post-treatment (p < 0.05).

To learn if global rFCN dynamics are functionally meaningful, we compared FCN between controls and patients. CC, CPL and small worldness (SW) increased at around 300 ms in the network in both groups which corresponds to the cognitive stage of the P300 in VEP recordings^19^. In optic nerve damage patients, however, FCN had higher CC, longer CPL, but weaker theta-band SW organization during the different functional stages (Fig. 3). This indicates that neural processing during visual performance requires more steps in patients’ theta network (lower processing efficiency). Of note, we did not observe significant alterations in any other frequency networks.

**Figure 4:**
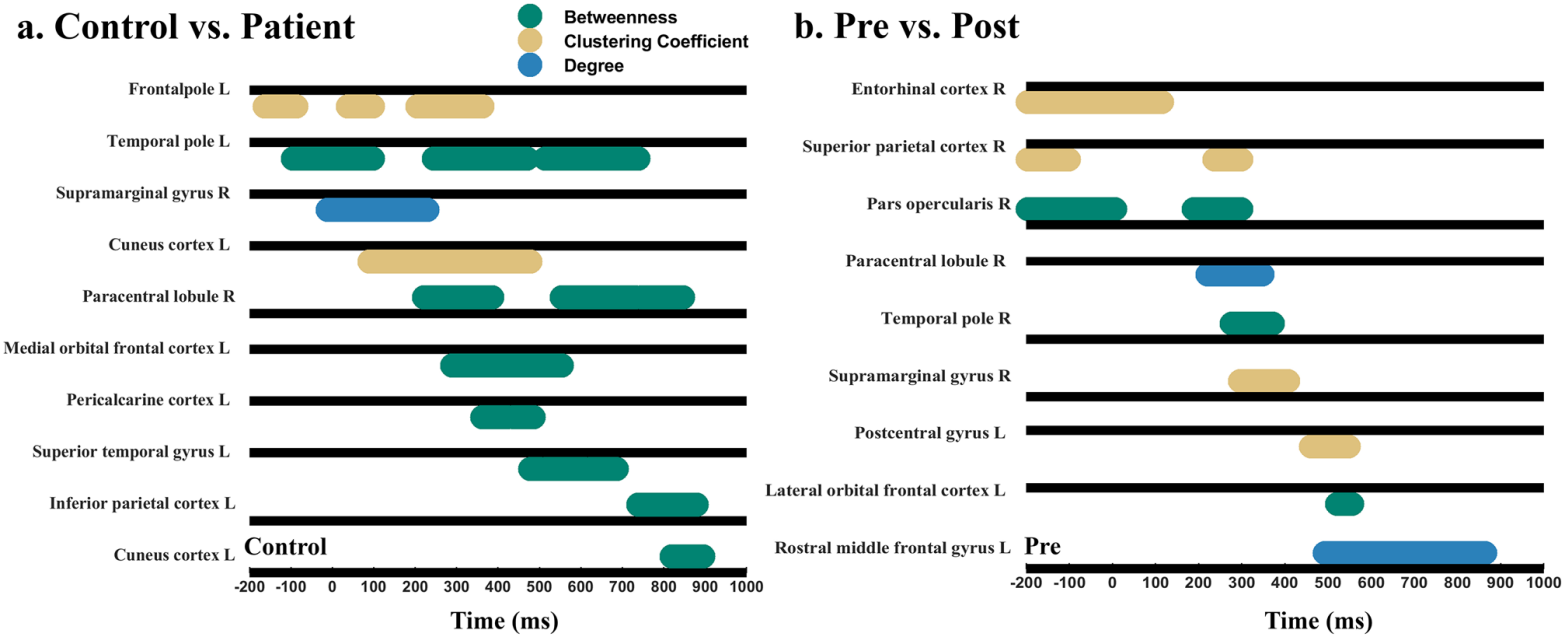
Significant node centralities as a function of the network state. Significant node centrality dynamics for different brain regions as a function of time in reference to stimulus onset (0 ms) during the visual detection task. The black line is the respective reference value for the control group (**a**) or the pre-rtACS group (**b**). The colored horizontal bars display time windows of significant differences between groups (p < 0.05) (green: betweenness; yellow: clustering coefficient; brown: degree). Whereas activation increases are plotted above the respective reference line, activation decreases are plotted under the line. (**a**) The differences of local hub properties between controls and patients which were mainly observable for “betweenness centrality” which was higher in controls than in patients. But three non-hub regions in patients (hub score < 2) show significantly stronger betweenness than controls. Other non-hubs of patients showed significantly weaker local activation in multiple node centralities. (**b**) Effects of rtACS neuromodulation on rFCN dynamics. Brain regions showing significant differences of local FCN attributes after rtACS treatment (p < 0.05). While node centralities of some brain regions increased following rtACS neuromodulation, others decreased.

Node activity changes over time were found in node degree, node betweenness, node closeness and node clustering coefficient, with transient engagements of different brain regions in *space* as a function of *time*. Compared to controls, patients had local disruptions in both hub and non-hub regions: three hubs showed transiently lower betweenness during visual processing in patients: left medial orbital frontal cortex at around 290–554 ms (tmass = 88, p = 0.02), left superior temporal gyrus [478, 686] ms (tmass = 67, p = 0.01), and left temporal pole in time ranges of [− 94, 97] ms (tmass = 63, p = 0.01), [246, 470] ms (tmass = 97, p = 0.004), and [514, 738] ms (tmass = 77, p = 0.007) (Fig. 4a).

Regarding non-hubs, node activities of five regions were transiently lowered in patients, reflecting less synchronization compared to normal visual processing. Three regions located in the visual network (VN), including left cuneus cortex (CC: [100, 500] ms, tmass = 136, p = 0.01), left lateral occipital cortex (CC: [− 195, 5] ms, tmass = 68, p = 0.02) and left pericalcarine cortex (Degree: [82, 345] ms, tmass = 88, p = 0.02; Betweenness: [361, 486] ms, tmass = 32, p = 0.03), two regions were located in attention network (AN) and default mode network (DMN) respectively, including right supramarginal gyrus (Degree: [− 10, 230] ms, tmass = 82, p = 0.02), and left frontal pole (CC: [− 163, − 87] ms, tmass = 22, p = 0.04; [37, 97] ms, tmass = 17, p = 0.05; [206, 361] ms, tmass = 47, p = 0.02) (Fig. 4a). But there were also three non-hub regions in patients which showed transiently stronger betweenness in patients during visual processing in the right paracentral lobule ([222–385] ms, tmass = − 50, p = 0.02; [550–730] ms, tmass = − 68, p = 0.01), left inferior parietal cortex ([738–878] ms, tmass = − 48, p = 0.01) and left cuneus cortex ([818–894] ms, tmass = − 25, p = 0.05) which signifies ‘compensation’ (Fig. 4a).

### Probing Brain Spacetime rFCN using rtACS neuromodulation in patients

More detailed FCN dynamics can be obtained when the time-vector is considered which shows great variability. Two hubs were transiently strengthened in degree or betweenness after rtACS in patients: the left superior temporal gyrus (Degree: [794, 870] ms, tmass = 26, p = 0.03), which had a lower betweenness in the attention network than controls, and the right temporal pole (Betweenness: [278, 369] ms, tmass = 36, p = 0.03), showing a synchronous enhancement, while before rtACS, betweenness of the left temporal pole was weaker. Another hub, the right entorhinal cortex, showed a significant decrease in its clustering coefficient (CC: [− 195, 113] ms, tmass = − 137, p = 0.001). In sum, rtACS modulated local connection patterns of hubs.

After rtACS, node centralities were also changed in several non-hub brain regions. Specifically, four non-hub nodes in the attention network were more enhanced as revealed by multiple node centrality metrics: left pars triangularis (Betweenness: [− 195, − 127] ms, tmass = 24, p = 0.04; [900, 1000] ms, tmass = 42, p = 0.004), right pars opercularis (Betweenness: [− 195, 5] ms, tmass = 78, p = 0.02), left rostral middle frontal gyrus (Degree: [494, 862] ms, tmass = 145, p = 0.01) and right supramarginal gyrus (CC: [298, 405] ms, tmass = 71, p = 0.005). In addition, CC was transiently increased after rtACS in the right pericalcarine cortex ([73, 169] ms, tmass = 43, p = 0.04) in visual network and left precentral gyrus (Betweenness: [602, 638] ms, tmass = 16, p = 0.05) and in sensory-motor network (Fig. 4b).

But there were also six transient non-hub deactivations after rtACS treatment: two were in the attention network, including right pars triangularis (Degree: [602, 786] ms, tmass = − 65, p = 0.02; [838, 942] ms, tmass = − 42, p = 0.03) and the right superior parietal cortex (CC: [− 195, − 102] ms, tmass = − 45, p = 0.001; [237, 297] ms, tmass = − 21, p = 0.03), and two in the default mode network, including left lateral orbital frontal cortex (Betweenness: [522, 554] ms, tmass = − 17, p = 0.04), right lateral orbital frontal cortex (Betweenness: [454, 562] ms, tmass = − 45, p = 0.02) and two in the sensory-motor network, including right paracentral lobule (Degree: [221, 345] ms, tmass = − 52, p = 0.02), and left post-central gyrus (CC: [462, 546] ms, tmass = − 34, p = 0.03) (Fig. 4b). Thus, rtACS neuromodulation altered patients’ ability to transiently synchronize oscillatory activity throughout the brain, affecting the reorganization of the attention network, visual network, sensory-motor network and default mode network. In addition, some FCN transients could be identified, showing that rtACS can normalize FCN dynamics (Fig. 3). Specifically, rtACS led to significantly enhanced SW at baseline in patients (at the time just before stimulus presentation), and in the cognition and execution stage. rtACS enhanced the theta, low and high alpha networks, but it did not alter the beta network. There were no such activation changes in the placebo group. We conclude that neuromodulation with rtACS enhanced the balance between integration and segregation of global rFCN.

### Correlation between FCN parameters and visual performance

**Figure 5:**
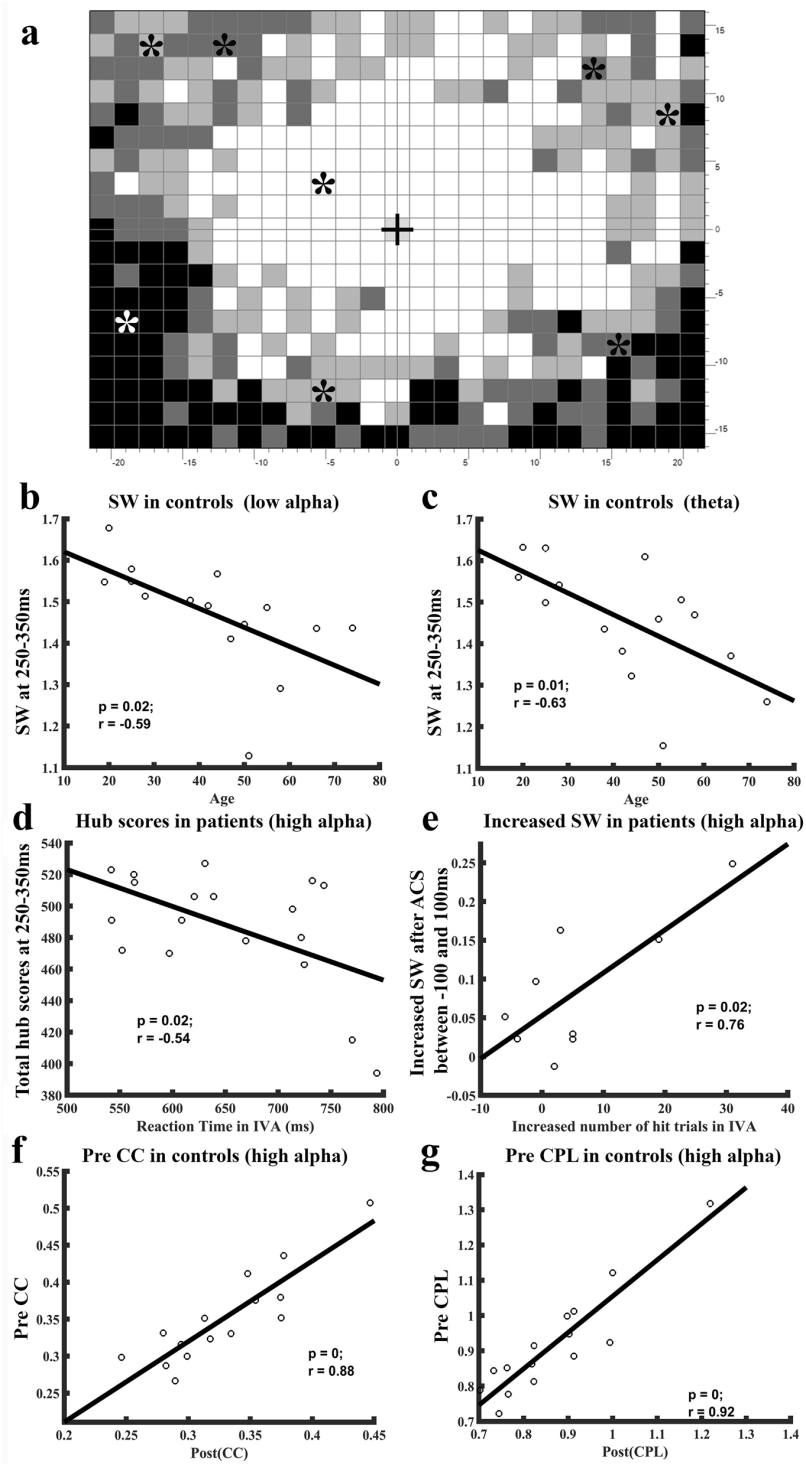
Visual detection performance in patients correlate with network metrics. (**a**) Typical perimetric visual field chart with central fixation (black cross) obtained from a patient showing individual placement of the stimulus positions (*stars). Grey levels represent percentage of responses detected in the respective region. One stimulus position was located in a white (intact) region of the visual field, six in grey areas of “partial” vision, and one in the blind region (black). (**b**–**g**) In normal subjects, small worldness (SW) was negatively correlated with the subjects’ age. Older subjects had weaker SW attributes during 250–350 ms following stimulus presentation in low alpha (**b**) and theta (**c**) rFCN. In optic nerve damage patients, higher total hub-score at 250–350 ms time window were correlated with the shorter reaction time (**d**). In patients after rtACS treatment, the increased SW during − 100 to 100 ms was positively correlated with the increased number of hit trials in the intact visual field sector (**e**). In addition, the maximum of clustering coefficient (**f**) and characteristic path length (**g**) in the post-task (250–650 ms) network were both positively correlated with the pre-task (100 ms before stimuli) network state. This means that ACS optimized the rFCN of patients, i.e. better vision was associated with a higher hub score and increased small worldness. And brain states at the time of visual stimulus onset had an influence on final task performance.

To explore if FCN attributes correlate with vision performance, we calculated Pearson’s correlation of FCN metrics with age, reaction time and number of hit trials in both healthy controls and patients with optic nerve damage. We found that better visual detection performance was associated with transiently elevated hub scores (synchronization) and increased SW (greater balance of integration and segregation). Specifically, the transiently higher hub scores in patients at 250–350 ms correlated with faster reaction times*,* but this correlation was not present in normal subjects. After rtACS treatment, increased SW during − 100 to 100 ms (baseline and early sensory processing) was also positively correlated with the increased number of visual detections (hits). In normal subjects, SW was negatively correlated with age (Fig. 5b–e), but SW was not associated with reaction time or number of hit trials. Follow-up visual field tests were conducted after 8 weeks treatment and were found to be stable for at least 2-months. This result demonstrates the stability of the visual field parameters and their correlation with our rFCN metrics. In addition, it is known that metrics of human brain networks have a good test–retest reliability in the relevant graph-theoretic analysis and are rather constant over time, highlighting the stability and reliability of graph metrics^20^.

We further analysed the pre-task network status and found a significant correlation between pre and post-task performance on multiple network parameters. It revealed that the maximum of global parameters in the post-task network were positively correlated with the pre-task network state in different frequency bands, including low alpha, high alpha, and beta, for both the control and patient groups (Fig. 5f,g). Perception in the brain can be viewed as a highly selective process, where top-down stimuli processing can create dynamic predictions about forthcoming events in stimulus-evoked and ongoing temporal activity^21^.

This supports the notion that brain states at the time of visual stimulus onset influence final task performance, providing remarkable insights into brain state-dependent, yet task-related, dynamics of rapid brain network reorganization.

## Discussion

We visualized for the first time behaviorally meaningful brain FCN dynamics in the millisecond resolution during visual processing and recovery as a function of 3D-topology “Brain-*Space*” in the 4th dimension of “*Time*”, i.e. 4D-“*Spacetime*”. Rapid and transient FCN hub and node interactions evolved and dissolved within milliseconds, and this rapid FCN plasticity was associated with different phases of visual processing, co-varying in patients before and after rtACS neuromodulation-induced recovery.

Our analysis of rapid FCN plasticity extends prior studies of resting-state networks, which is the current standard to characterize the physiological basis of neurological function and behavior^22^. Using graph theory we uncovered complex network features such as global clustering and efficiency, small-world attributes, and heterogeneous degree distributions^23^. FCN alterations during the resting state were reported in different neurological and psychiatric disorders, including depression^24^ and partial optic nerve damage^17^ which revealed lower FCN coherence, less dense clustering, loss of small-worldness, and long-term reorganization. But unlike such resting-state studies, rFCN analysis can inform us of rapid topological centrality and node activity changes as a function of time, indicating how fast the brain network can change in response to visual stimulation and in which time range such FCN plasticity happens.

We plotted 8 ms EEG epochs to quantify fast and transient FCN plasticity in normal subjects and in patients with visual system damage. Using ERNA^16^, we now showed that “FCN transients” are associated with different phases of visual processing in normal subjects and are altered in patients before and after vision recovery. Apparently, rFCN reorganization in patients is not as flawless as in normal subjects, especially in hub regions and the visual network. But rtACS is able to induce rFCN recovery of hubs and reorganization in the attention and vision network. However, patterns of neural FCN phase synchronizations are rather complex, in (brain space) location, strength and timing.

We identified 20 hubs in control subjects, of which 10 belong to the default mode network (DMN)^25^, a rather stable network of anatomical regions believed to support different functions such as internal modes of cognitive^26^ or episodic memory processing^27^. Six hubs belong to the salience network (SN), which plays a key role in attention and the detection of behaviorally relevant stimuli by way of coordinating neural resources^28^. While our study confirms that DMN hubs are rather stable in location, we now unveiled that their strength, and the connections between them, are highly dynamic, establishing and dissolving during visual processing in the millisecond range. Such transient DMN hub activations possibly provide a network-state reference frame for temporal binding of visual processing with other, non-visual processes such as emotions, expectations, visual memories etc. Clearly, the DMN is not a passive “internal” network, but it influences, or is influenced by, sensory, cognitive or motor execution tasks. This is in line with the argument that the DMN plays a role in task relevant processing^29^.

The question arises why hub scores are stable even though their connections rapidly change. As Fig. 2 and the Supplementary Video S1 show, the inter-hub connections among hubs are highly dynamic as shown with 8 ms resolution. Thus, although the connection between two hubs may appear to have disappeared, each hub quickly establishes connections with other hubs again. This rapid presence or absence of connections contributes to the hub-score, and the sum of all connections can, in fact, remain relatively stable at a resolution of 8 ms. Furthermore, we only analyzed stronger connections of our hubs, but hubs can also have many weaker-type connections as well; it is the sum of all connections -stronger or weaker ones- that contribute to the hub score.

These 20 hubs play a key role not only in normal visual processing but also in patients with visual system damage. Here, three hubs were disrupted in their betweenness centrality, and some “non-hub” regions had weaker local activations in the visual and attention network. In view of the special role of frontal-occipital network interactions in visual processing^17^, we believe that such network disturbances cause interruptions during visual related cognition due to FCN reorganization in key hubs, even if these brain regions are anatomically “intact”. This might help explain subtle visual disturbances or pseudo-hallucinations in low vision patients^30^. Furthermore, two non-hub regions that belong to the visual and attention network showed stronger betweenness during visual processing in patients: the left inferior parietal cortex and left cuneus cortex ROIs. On one hand, this illustrates the disruption of hub regions and peripheral visual and attention networks in patients’ rFCN, but -on the other hand- it also documents compensation in non-hub regions to adapt to the loss of interactions in patients during visual processing.

On the global level, rFCN in patients showed a greater degree of local integration with higher functional specialization (high clustering coefficient) than controls. This suggests reduced flexibility (i.e. greater stability or rigidity), possibly as a consequence of the loss of long-range interactions. Our findings do not agree with resting-state FCN studies in other brain disorders where decreased local integration was interpreted as evidence of local clustering decreases in Parkinson patients^31^ and in alcohol dependency^32^. A possible explanation for this discrepancy is that optic nerve damage leads only to *functional*, not *structural,* deafferentation.

Increased clustering between the pairs of neighbors in patients’ rFCN also signifies longer path length and poorer small-worldness in rFCN, a sign that neural information transfer needs more steps (greater processing cost), reducing processing speed. This interpretation fits our observation that longer reaction times correlate in patients with lower hub scores in many (but not all) ROIs. Increased path length was also reported in Alzheimer’s Disease^33^ and Depression^34^. In sum, patients with optic nerve damage suffer a functional imbalance of specialization versus integration. These *Spacetime* disturbances reduce neural processing efficiency at both local and global levels throughout the brain.

To learn if changes on rFCN are associated with different behavioral states, we studied the dynamics of rFCN following neuromodulation using rtACS treatment to  evaluate if this impacts vision recovery. rtACS is a non-invasive method which can modulate ongoing brain activity rhythms: it enhances alpha activity in normal subjects^35^ and in patients with optic nerve damage^36^. And it induces “after-effects” that outlast the stimulation period^37^. Repeated treatment with rtACS modulates resting-state FCN re-synchronization, enabling more effective information transfer both in normal subjects^38^ and in patients^17^. Our rFCN findings confirm this conclusion and show that rtACS normalizes integration within subsystems and improves small world organization at rest (baseline), at early sensory and at late cognition stages in patients. The peripheral attention network and visual network also showed significant enhancement after rtACS, and the default mode network and some regions of visual network reorganize in their hub score dynamics during vision processing. In sum, we observed rapid FCN plasticity both in normal subject and in patients as supported by many transient FCN patterns especially in the alpha-rhythm. In fact, visual performance correlated significantly with greater alpha network synchronization, and both reaction time and visual detection improved after alpha rtACS stimulation, which, in turn, significantly correlated with alpha PLV networks. Therefore, we interpret visual improvement as a sign that alpha frequency oscillating networks induce activation, not inhibition, of visual processing.

The alpha rhythm has long been known as a fundamental mechanism of perception and cognition, affecting multiple top-down cognitive processes^39^. Therefore, we focused our analysis on the alpha PLV networks. While alpha oscillations are functionally distinct, we cannot tell if they serve an inhibitory or active purpose. In some tasks alpha oscillations may support inhibition of task-irrelevant neuronal processing on amplitude dynamics in several EEG and functional MRI studies, yet others suggest that alpha phase dynamics support activation in task-relevant functions like attention^40^, working memory process^41^ and executive functions^42^.

While our research focused upon alpha frequency network modulations, we recognize that neural processing involves simultaneous oscillations in multiple frequencies, not just alpha. For example, beta oscillations represent coordination and adaption between neurons in the motor system^43^, and theta oscillations largely serve to structure recurrent interactions of neurons during working memory^44^.

Other types of phase synchronization across frequencies exist, like cross frequency coupling (CFC)^45^ to integrate neuronal activity across different spatial and temporal scales. The relation and statistical dependence across frequencies awaits further experimental evidence due to the complexity of the different varieties of CFCs, like phase–amplitude CFD, phase–phase CFC and amplitude–amplitude CFC.

Nevertheless, our observation of functionally relevant millisecond dynamics of FCN has broad implications of how neural information is synchronized in the dimensions of neural 3D-*space* and the 4th dimension of *time* in visual processing and recovery. Specifically, we showed that nodes and their connections (i) can be monitored at millisecond resolution, (ii) they vary in strength, stability and dynamics over time, (iii) they are disorganized in patients, and (iv) neuromodulation with rtACS modulates temporal processing of rFCN which correlates with vision recovery. In analogy to A. Einstein’s principle of spacetime where in the universe *space* and *time* are tigtly intertwined, we propose that “Brain Spacetime” is a fundamental principle of the human mind, where "brain space" and time are also tightly intertwined. Brain spacetime is relevant not only for normal vision cognition and vision restoration, but also critical for our basic understanding of normal and abnormal human perception, cognition and overt behavior.

## Materials and methods

### Participants

Following local ethics committee approval, 22 patients with optic neuropathy (eight females) and 15 healthy controls (seven females) were recruited for this clinical study. Patients were randomized and double-blind to the rtACS (n = 12) and placebo group (n = 10). The ages of the controls and patients were comparable (46% and 44%, respectively in the 40–60 years age group).

Causes of optic nerve damage were anterior ischaemic optic neuropathy/AION (N = 6), post-inflammatory (N = 4), and various other causes. Inclusion criteria were residual (patients) or normal vision (controls). Exclusion criteria were instable intraocular pressure (> 27 mmHg) or history of epilepsy, heart pacemakers, photosensitive epilepsy, psychiatric diseases (schizophrenia etc.), high blood pressure or diabetes.

### Visual field diagnosis

Visual fields were monitored with computer-based high-resolution campimetry (HRP)^46^. Briefly, subjects were seated 42 cm in front of a monitor and asked to fixate a central fixation spot while responding to 475 successive, white target stimuli presented at random locations.

As shown in Fig. 5, the visual field was divided into three functional areas as defined by detection rate: intact visual field sector (IVA), shown in white, where the subject detect correctly 3/3 stimuli in the same location, partly defective regions (“areas of residual vision”, ARV), where 1–2 of 3 stimuli were detected, and blind visual field areas without a response. Eight positions were then individually selected per patient for additional visual evoked potential (VEPs) testing: one in IVA, six in the ARV, and one in the blind area (Fig. 5a).

### EEG recording of visual evoked potentials

Visual evoked potentials (VEPs) were collected with an EEG amplifier (Brain Products, Munich, Germany) with 53 sintered Ag/AgCl electrodes mounted in an Easycap (Falk Minow Services, Munich, Germany) according to the 10–10 system, referenced to nose-tip with ground electrode at Fz and Cz. VEPs were recorded monocularly on patients’ damaged eye and the same eye for matched normal subjects. The VEP stimulus (400 ms) was either a circle (1° diameter) or a square (1 × 1°) which had to be acknowledged by corresponding bar press. The stimuli were presented at eight different locations on the basis of HRP for 400 ms, and at each location, 180 trials were performed for each subject (random inter-stimulus-interval of 1300–1700 ms). Two patients in the rtACS group dropped out without EEG after HRP. One patient in the rtACS group and one patient in the placebo group had to be excluded because of the limited number of detected trials (< 50), leaving nine patients in the rtACS group and nine in the placebo group. On average 140 ± 2 (s.e.m.) trials were analyzed for each subject/condition.

### Repetitive transorbital alternating current stimulation (rtACS)

Non-invasive brain current stimulation is now a well-established method to alter functional connectivity networks^6^. rtACS is the preferred method to modulate both normal and abnormal visual functions and functional connectivity^37^. We treated our patients with established protocols^18^ using four stimulation electrodes (sintered Ag/AgCl ring electrodes, Easycap, Germany) placed near the eyeball. Current bursts in the alpha-frequency range (amplitude < 2000 μA peak-to-peak) were applied daily for 10 days (15 min per eye). rtACS is both effective, safe, and well tolerated^37^.

The study was approved by the ethical standards committee for human subjects (institutional). All participates were treated in accordance with the Declaration of Helsinki and signed a written informed consent.

### EEG pre-processing and source reconstruction

EEG epochs were filtered to 1–100 Hz, notch 50 Hz FIR filter, down sampled to 250 Hz and average re-referenced and time-locked − 0.8 to 1.7 s. Epochs with artifacts and noisy channels were removed with independent component analysis (ICA). For each subject, we selected 15 ± 3 components and projected them back into sensor space.

Following EEG pre-processing, source-localized activities were obtained by applying geometry and electrical conductivity of the tissues in the head using a forward model to estimate how neuronal currents propagate from source regions within the brain to the EEG sensors (electrodes). Here, the anatomical Colin27 head template was used as a common geometric model. The forward model was calculated using the boundary element method (BEM)^47^ to describe electrical current properties of the head, and source current distributions were applied to estimate the weighted minimum norm estimate (wMNE)^48^. wMNE is a classical EEG inverse transformation to overcome the limitations of preference in superficial sources, but it can also induce deep generator activities with high accuracy. The dipole orientation was constrained perpendicular to the cortex. The average of all dipoles belonging to the same region was calculated representing the activity of each area. In this way, sensor signals were projected onto an anatomical framework so that source-reconstructed neuronal activities could be obtained for 68 cortical regions of interest (ROIs; 34 per hemisphere) and we computed the mean voxel time series for each ROI as defined by Desikan–Killiany^49^.

### Functional connectivity network construction

Source data were then digitally filtered (band pass filter 3.9–30 Hz) using Morlet wavelets. A sliding window fixed at 211 samples (844 ms) irrespective of the frequency was used to analyse the signal. The time–frequency representation of the data was thus estimated from a minimum frequency of 3.9 Hz with three cycles to a maximum frequency of 30 Hz with 11.4 cycles. The data with 8 ms and 0.7 Hz resolution were then generated. In this way, instantaneous measurements of EEG data were decomposed into temporal and spectral bands.

Phase locking value (PLV)^50^ were used to estimate the functional connectivity between all pairwise ROI combinations. PLV represents synchronies, commonly describing long-range synchronization patterns between widely separated brain regions which was computed as

1 $${PLV}_{\left(f,t\right)}=\frac{1}{N}\left|{\sum }_{n=1}^{N}exp\left(i\left({\Delta \varphi }_{n}\left(f,t\right)\right)\right)\right|,$$

here, ${\Delta \varphi }_{n}\left(f,t\right)$ denotes the phase difference between ROIs for frequency f and time point t. N is the number of trials, and ǁ the absolute value. The PLV measures the inter-trial variability of the phase difference at t. It ranges between 0 and 1, where PLV close to 1 shows that the phase difference varies little across the trials (“phase locking”). A threshold of 0.29 was applied to convert full PLV values into edges of weighted network.

### Network topology metrics

To describe *Brain Spacetime*, we used graph theory to mathematically characterize brain rFCN^51^. Graph metrics included clustering coefficient (CC), characteristic path length (CPL), small-worldness (SW) index and Hub topography. CC quantifies segregation of neural processing, capturing the presence of clusters within the network. *CPL* represents the average minimum path length between all pairs of nodes in the network.

Hubs can be viewed as local topological “centers” of synchronization, and the “Hub scores” represents their relevance for a given function (here: vision). The hub score varies from 0 to 4, where the top score of 4 is reached when the following criteria of centrality are fulfilled: high weighted node degree, node betweenness, and node closeness, but low node clustering coefficient^52^. This means that the higher the hub score, the higher up is the node in the FCN hierarchy^53^. A node receives a score of 1, if it ranks in the top 20% of nodes with highest degree in one of the four node criteria. In our study, a node was identified as a “hub” only if the hub score was 2 or higher and lasting > 50 ms which is at the threshold of temporal discrimination^15^. Otherwise, the node was termed “non-hub”.

### Statistical analysis

Both study groups were statistically comparable in age and gender. Statistical analyses of network metrics were calculated with cluster mass permutation tests. Because the EEG signal was sampled and analyzed multi-dimensionally (time and frequency bands), we considered the multiple comparisons problem (MCP)^54^ with appropriate alpha-adjustments. The cluster mass permutation test^55^ was used to control family-wise error rate (FWER) at some critical alpha level which solves the MCP. Here, a false alarm rate of p = 0.05 was chosen and the cluster inclusion threshold was set at p = 0.025.

## Supplementary Information

Supplementary Video 1.

## Data Availability

The data that support the findings of this study are available from the corresponding author, upon reasonable request.

## Abbreviations

FCN: Functional connectivity networks
rtACS: Repetitive transorbital alternating current stimulation
IVA: Intact vision area
